# Cellular Density Effect on RGD Ligand Internalization in Glioblastoma for MRI Application

**DOI:** 10.1371/journal.pone.0082777

**Published:** 2013-12-27

**Authors:** Damien Moncelet, Véronique Bouchaud, Philippe Mellet, Emeline Ribot, Sylvain Miraux, Jean-Michel Franconi, Pierre Voisin

**Affiliations:** 1 CRMSB, UMR 5536, University Bordeaux Segalen, CNRS, Bordeaux, France; 2 INSERM, University Bordeaux Segalen, Bordeaux, France; Ospedale Pediatrico Bambino Gesu', Italy

## Abstract

Cellular density is a parameter measured for glioma grade and invasiveness diagnosis. The characterization of the cellular density can be performed, non invasively, by magnetic resonance imaging (MRI), since, this technique displays a good resolution. Nevertheless MRI sensitivity is critical. Development of smart contrast agents appears useful to increase MRI signal to noise ratio (SNR). Tumor invasiveness is correlated with high expression of integrins that can be targeted by RGD motif. In this study, MRI contrast agents or fluorescent probes linked to RGD-peptides were used, in a glioma model, to assess the relation between RGD uptake/signal improvement/cell density and consequently tumor invasiveness.

Experiments were performed in vitro with U87-MG glioma cells. Flow cytometry and microscopy experiments with RGD and iRGD-alexa488 demonstrated that cell internalization was dependent on cell density. The internalization involved a clathrin-dependent endocytosis. Cytoskeleton and particularly the microtubules were concerned. Actin filaments played a minor role. The internalization was also dependent on the glycolysis and the oxidative phosphorylations. The cellular density modulated the importance of the endocytosis pathways and of the metabolism but not the cytoskeleton contribution. The internalization of the RGD-peptide associated to gadolinium chelate increased the SNR of U87 cells. Moreover, following the cell density augmentation, the SNR increased with a low amplitude but a trend was clearly determined. In conclusion, RGD-peptide internalization appeared, in vitro, as a marker of cellular density. In perspective, the combination of these peptides with contrast agents associated to more sensitive MRI techniques could improve the MRI signal allowing the characterization of cellular density for tumor diagnosis.

## Introduction

Glioblastoma multiform (GBM) is the most frequent primary malignant brain tumor in adults. Classification and prognosis of malignant glioma are based on histologic features and imaging. One of the parameters assessed by histology is the cellular density within the glioma [1]. Cellular density correlates with the grade and the invasiveness of this tumor. Consequently, cell density quantification in the various tumor areas give information for diagnosis. However, histology studies involve biopsies that are invasive for the patient. Thus, magnetic resonance imaging (MRI) can be helpful to measure the tumor cellular density in a non invasive manner [2]. MRI is well adapted since this technique displays a good spatial resolution although it appears limited by a low sensitivity.

Thus, the frame of our study is to evaluate the possible use of targeting agents displaying properties dependent on tumor cell density. Combination of these agents to contrast media could balance this lack of MRI sensitivity and allow the acquisition of non invasive functional data that would help to appreciate the invasiveness of tumors with more accuracy. Among the proteins over-expressed by GBM, integrins represent potential targets for diagnosis and therapies [3].

Integrins have critical functions in angiogenesis, cell invasion and migration. αvβ3 and αvβ5 integrins recognize Arg-Gly-Glu (RGD)-motif containing peptides. Recently Sugahara et al. [4] developed the tumor-homing «internalizing RGD» peptide (iRGD, CRGDK/RGPD/EC) that presents RGD and C-end Rule (CendR, R/KXXR/K) motifs [4]. The CendR motif is not active unless it occupies a C-terminal position in the peptide after cleavage, recognizing neuropillin-1 and facilitating the penetration into the tumor of iRGD and co-administered drugs. However, the mechanisms involved in iRGD cell internalization are not well known. Since their discovery, RGD-peptides became useful tools for the targeting of imaging agents and/or drugs to glioma areas expressing αvβ3 and αvβ5 [5]. For instance, the improvement of the MRI signal to noise ratio (SNR) of the tumor vasculature is possible by cross-linking iron oxide (CLIO) nanoparticles or ultrasmall superparamagnetic iron oxide particles (USPIO) with RGD-peptides [6] [7]. iRGD-peptides are also used for imaging [8], therapy [9] and known to increase the penetration of nanoparticles inside the tumors, even when iRGD and nanoparticles are not chemically conjugated [10].

Moreover, imaging and therapy depend on the intracellular concentration of the RGD contrast agents and their intracellular trafficking. Integrin internalization is mediated by endocytosis and can implicate clathrin [11], caveolin [12] dependent endocytosis and macropinocytosis [13]. Cytoskeleton especially actin filaments, microtubules and intermediate filaments like vimentin are also involved [14]. All these processes are energy dependent. ATP is a major energetic intermediate as phosphorus donor for metabolic and signaling pathways. ATP can be supplied from various metabolism pathways like glycolysis, oxidative phosphorylations, β-oxidation of fatty acids or oxidative degradation of amino acids.

The aim of this study was to characterize the regulation of the RGD-peptides uptake by cellular density. The second goal was to assess, in vitro, how RGD ligands, conjugated to gadolinium, could be useful as MRI contrast agents to evaluate the cellular density within a glioma.

## Materials and Methods

### Cell culture

Human GBM cell line U87-MG from the American Type Culture Collection (ATCC, LGC standards, Middlesex, UK) was cultured in Dulbecco's modified eagles medium (DMEM, Gibco Corp) supplemented with 10% fetal calf serum (FCS, Gibco Corp), in a humidified atmosphere with 5% CO2 at 37°C. The characterization of the metabolism contribution was performed with DMEM without glucose and pyruvate (DMEM, Gibco Corp). Glucose or pyruvate were added when necessary at the concentration of 1 g/L and 0.11 g/L , respectively.

### Labeling of E-[c(RGDfK)2] or iRGD with alexa fluor 488

E-[c(RGDfK)2] (Peptides International) and iRGD (Tebu-bio) were bound to amine reactive alexa fluor-488 Carboxylic Acid, Succinimidyl Ester, mixed isomers (Molecular Probes, Invitrogen). Alexa fluor-488 was solubilized in 200 µL DimethylSufoxide (DMSO) and added to 1 mL RGD or iRGD at 1 mg/mL in 0.5 mM Hepes at pH 8.3. After 2 h at 25°C, the peptide was separated from the free fluorophore using a G10 column (Sigma-Aldrich) (length:30 cm, volume: 241 cm^3^) in water running at flow rate of 0,5 mL/min. Concentrations of fluorescent RGD or iRGD were estimated by spectrophotometry at 519 nm. Peptides were stored at −20°C.

### Labeling of E-[c(RGDfK)2]-DOTA with Gadolinium

The synthesis of RGD-gadolinium chelate was performed by mixing E-[c(RGDfK)2]-DOTA (5 mg/mL, Peptides International) with GdCl_2_ (3 mg, Sigma-Aldrich) in Hepes (1 mL 0.5 mM pH 7) at 25°C during 2 h. Then the excess of free GdCl_2_ was discarded using a G10 column as previously described.

### Flow cytometry

Uptakes of E-[c(RGDfK)2]-Alexa488 and iRGD-alexa488 by U87 cells were compared for different cellular densities using flow cytometry. Twenty four multiwell plates (Becton-Dickinson, 2 cm^2^/well), were pretreated with collagen (Sigma-Aldrich) (10 µg/cm^2^). Then the cells were seeded during 24 h either at densities ranging from 8.10^3^ to 10^5^ cells/cm_2_ or at three typical densities being in the lowest, the median and the highest values of the previously mentioned density range. The cells were incubated 1 h with the fluorescent RGD ligands (E-[c(RGDfK)2]-Alexa488, 2 µM; iRGD-alexa488, 15 µM). Then the medium was discarded and the cells were washed with Roswell Park Memorial Institute Medium (RPMI, Gibco Corp). Thereafter the cells were re-suspended in RPMI at 100 cells/µl and analyzed by flow cytometry (Guava easyCyte flow cytometer/counter, Millipore, λ_exc_: 488 nm, λ_em_: 525 nm).

### Contribution of subcellular organelles

The clathrin-mediated endocytosis and the caveola-dependent endocytosis were characterized by incubations with chloroquine (100 µM) and nystatin (30 µM), respectively. Contribution of macropinocytosis was studied following amiloride treatment (1 mM).

The consequences of the actin filament depolymerization were observed with cytochalasin D (cytoD) (20 µM). The microtubule targeting was performed using paclitaxel (10 µM), colchicin (7.5 µM) and nocodazole (30 µM).

The oxidative metabolism was studied with oligomycine (20 µM) and rotenone (10 µM).

All of these inhibitors and drugs were applied on the cells for 1 h simultaneously with the fluorescent RGD ligands. When specified, incubation of drugs begun 20 min before the incubation with the RGD ligands. All the reagents were from Sigma-Aldrich.

### Microscopy

Protocols for microscopy were identical to those for flow cytometry. Cells were seeded on glass coverslips after collagen treatment as described above. After the different sets of incubations the cells were washed with RPMI and fixed with paraformaldehyde (4%, 20 min). Nuclei were then labeled with DAPI (0.01 mg/ml). After three washes, the coverslips were mounted on glass slides and observed using a Leica DM5500 B (magnification 63×, λ_exc_: 480 nm, λ_em_: 530 nm and λ_exc_:360 nm λ_em_: 470 nm for alexa fluor-488 and DAPI respectively).

### MRI experiments

E-[c(RGDfK)2]-DOTA-Gd uptake by U87 cells was performed on cells platted at 7.10^4^ cells/cm^2^. After 24 h, the cells were then incubated for 4 h with 400 µM of E-[c(RGDfK)2]-DOTA-Gd or for 1 h with MnCl2 at 400 µM as positive contrast for the T_1_-weighted (T_1_-w) sequence. Then the medium was discarded and the cells were washed with RPMI. The cells were re-suspended at 2.10_6_ cells in 100 µL of phosphate buffer saline (PBS) for each condition and mixed with 100 µL of agarose 2% (Sigma-Aldrich).

MR images of cells trapped in agarose gel were acquired on a 4.7 T Biospec system (Bruker, Ettlingen, Germany). The system was equipped with a 6-cm diameter BG6 gradient system capable of 950 mT/m maximum strength. Imaging was performed with a birdcage resonator (35-mm diameter and 80-mm length) tuned at 200.3 MHz. 2D images were acquired with a T1-w sequence (FLASH sequence, TE/TR: 1,6/14 ms, flip angle: 60°, FOV: 25*25 mm, matrix:128*96, 512 averages, total acquisition time: 5 min 44 s).

Seeded at three densities, cells were incubated with E-[c(RGDfK)2]-DOTA-Gd at 400 µM for 4 h. The same protocol to prepare cells trapped in agarose gel was used. 3D images of the RGD internalization were acquired with a T1-w sequence (FLASH sequence, TE/TR: 1,5/12 ms, flip angle: 20°, FOV: 30*25*20 mm, matrix:128*128*64, 6 averages, total acquisition time: 9 min 49 s).

### Statistical analysis

All experiments were performed in duplicate and reproduced at least 3 times. The mean ± SEM was calculated for each experimental group and the statistical significance was evaluated with one-way Anova followed by Tukey's multiple comparison test. The results were considered significant for p<0.01 and p<0.05.

## Results

### MR imaging of U87 cells labeled with E-[c(RGDfK)2]-DOTA-Gd

**Figure 1:**
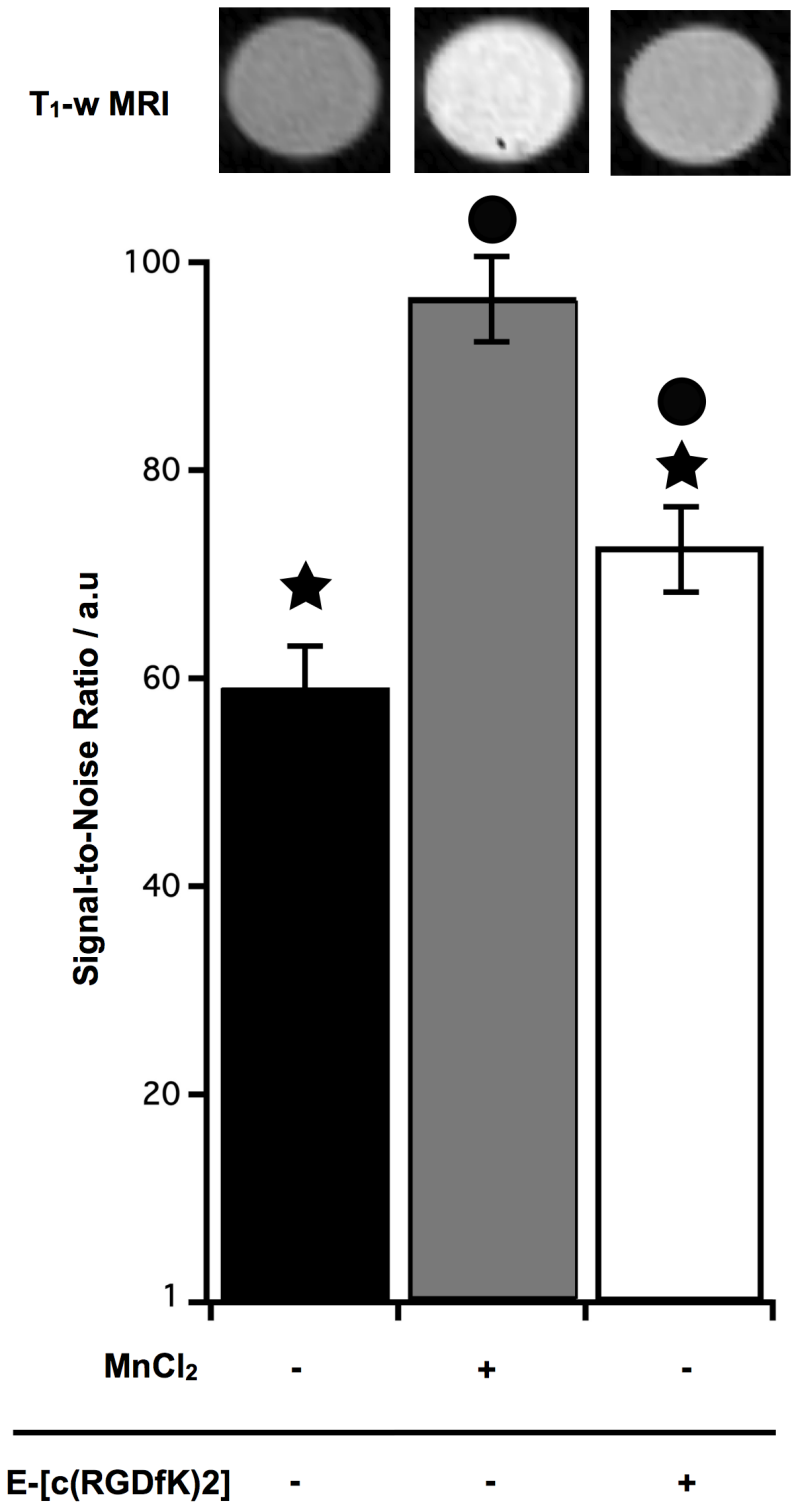
MRI signal of U87 cells after uptake of E-[c(RGDfK)2]-DOTA-Gd. U87 cells were incubated at 7*10^4^ cell/cm^2^, with E-[c(RGDfK)2]-DOTA-Gd or MnCL_2_ (400 µM during 4 h and 1 h, respectively). Applying a T_1_-weighted sequence (T_1_-w MRI), results with E-[c(RGDfK)2]-DOTA-Gd were significantly different from the unlabeled cells (★, p<0.05) and from the MnCl_2_ condition (•, p<0.05).

The suitability to use E-[c(RGDfK)2]-DOTA-Gd as MRI contrast agent to target the integrins expressed on U87 cells was studied. U87 cells, platted at medium density, were incubated 4 h with this RGD contrast agent or 1 h with MnCl_2_ at the same concentration (400 µM). MR images showed a SNR increase from 60 to 100 after labeling cells with the positive contrast control MnCl_2_ (figure 1). When the cells were incubated with E-[c(RGDfK)2]-DOTA-Gd, the obtained SNR was lower than the SNR for MnCl_2_ treatment (70 versus 100) but was more important than the SNR measured for the unlabeled U87 cells (70 versus 60). This increase in SNR resulted from either bound or internalized E-[c(RGDfK)2] peptides or from both. In order to settle the role of each phenomenon, we grafted a fluorescent probe on this peptide and on iRGD.

### Labeling of U87 cells with E-[c(RGDfK)2]-Alexa488 and iRGD-alexa488 was cellular density dependent

**Figure 2:**
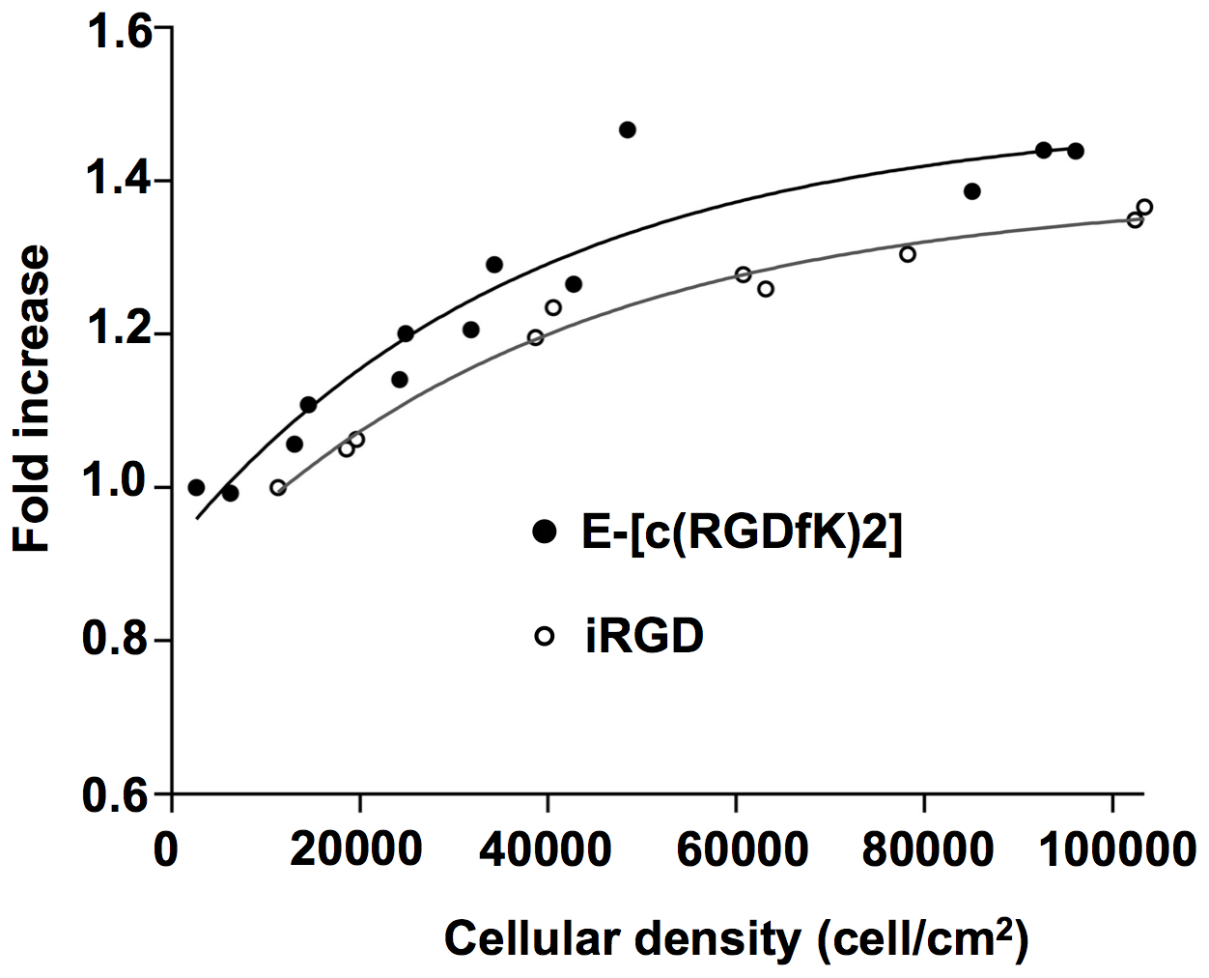
Cell density dependence of the U87 cell labeling by E-[c(RGDfK)2] or iRGD. The «fold increase» represents the normalized value of the median fluorescence intensity (MFI) observed for a given cell density compared to the lowest one (0.8*10^4^ and 10^4^ cell/cm^2^ for the E-[c(RGDfK)2] and the iRGD conditions, respectively). Experimental results were fitted following exponential curves of the type y = a + b × exp^−cx^ with a, b and c equal to 1.4853±0.064; −0.55979±0.0587; 2.5987×10^−05^±7.89×10^−06^ and 1.3862±0.0257; −0.52569±0.0249; 2.5936×10^−05^±4.42×10^−06^ for the E-[c(RGDfK)2] and iRGD respectively.

The uptake of E-[c(RGDfK)2] or iRGD was analyzed by flow cytometry for different cell densities (figure 2). U87 cells were incubated during 1 hour from 8*10^3^ to 10^5^ cells/cm^2^ with either E-[c(RGDfK)2] or iRGD, both being conjugated to the Alexa Fluor-488 probe. Cytometry results demonstrated that cell labeling increased with the cell density to reach a plateau following an exponential process. This increase was in a similar range reaching 40% and 30% for the highest densities when cells were incubated with the [c(RGDfK)2] and the iRGD, respectively.

### Sub-cellular location of E-[c(RGDfK)2]-Alexa488 and iRGD-alexa488

**Figure 3:**
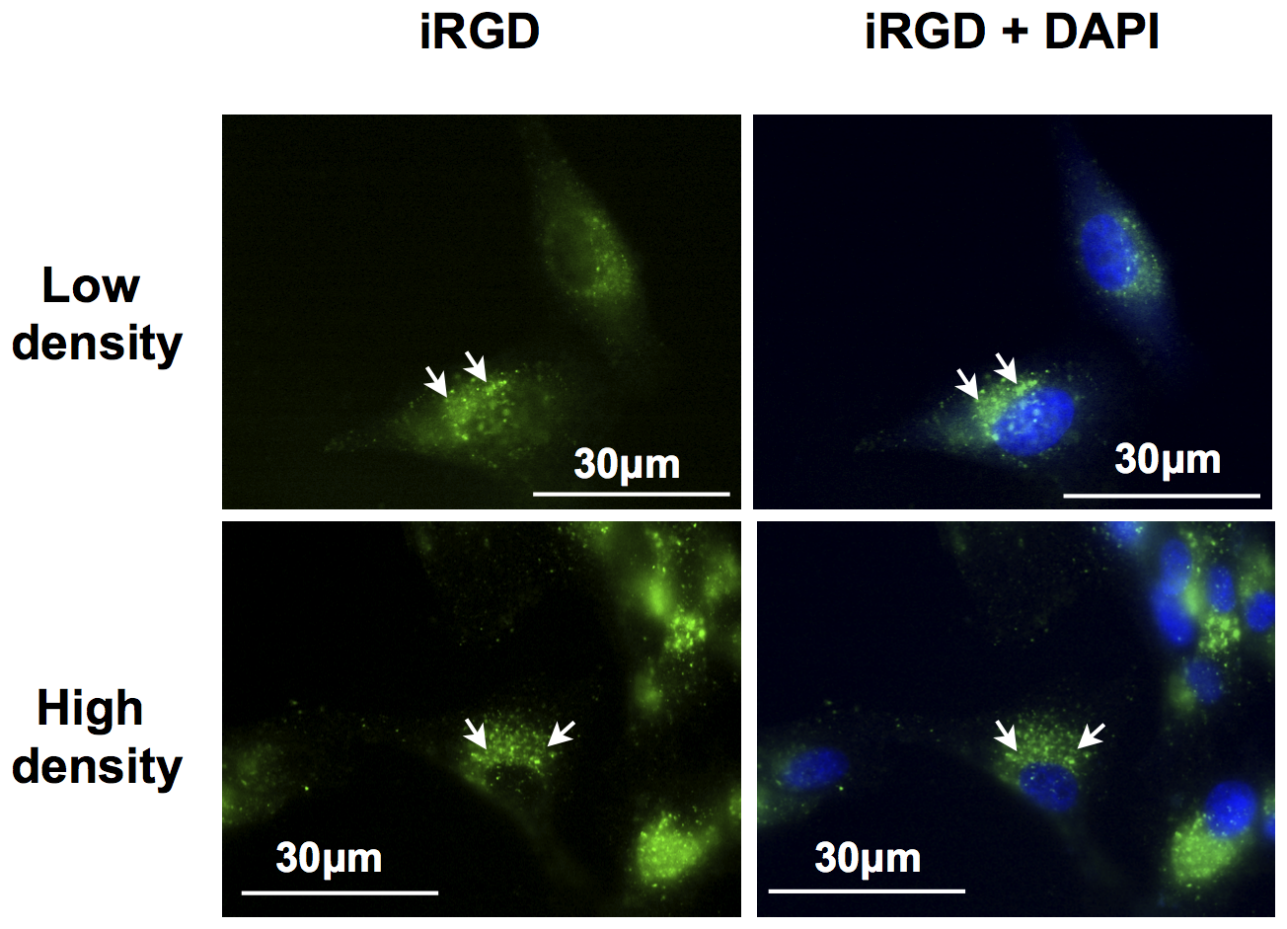
Observation by fluorescent microscopy of the iRGD-alexa488 internalization into U87 cells. U87 cells were incubated during 1h at 37°C with iRGD-alexa488 at 15 µM (green). Nuclei were stained with DAPI (Blue). Arrows showed vesicular accumulation of the fluorescence inside the cytoplasm. The cytoplasmic membrane labeling was not detectable.

For the different cellular densities, the contribution of the membrane bound versus the internalized RGD peptides on the signal was analyzed by fluorescent microscopy. U87 cells were incubated with the fluorescent iRGD according to the experimental conditions described in figure 2. As shown in figure 3, most of the signal appeared intracellular. Membrane labeling was undetectable suggesting that after 1 h of incubation, most of the iRGD ligand was internalized and concentrated in endosomal or lysosomal like vesicles. This fast accumulation occurred for all the studied cellular densities. Similar results were obtained with E-[c(RGDfK)2] (data not shown).

### Analysis of the endocytosis pathways of E-[c(RGDfK)2]-Alexa488 and iRGD-alexa488

**Figure 4:**
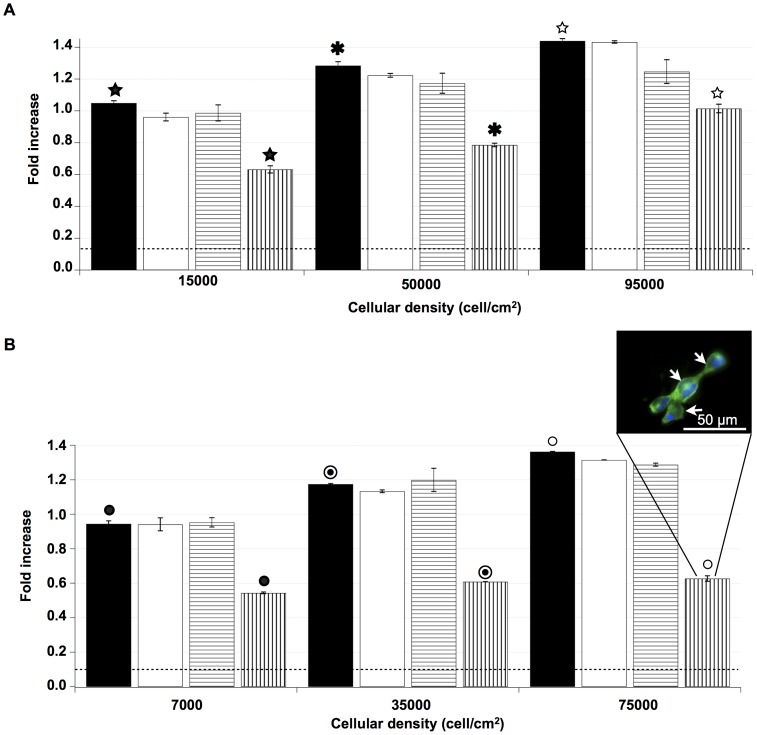
Cellular density effect on endocytsis pathways of E-[c(RGDfK)2] and iRGD. U87 cells were incubated with E-[c(RGDfK)2] at different densities (15000; 50000; 95000 cell/cm^2^) (A) or iRGD (7000; 35000; 75000 cell/cm^2^) (B).These densities were defined as low, medium and high density, respectively. Incubations were performed without inhibitor (▪), with nystatin (□), with amiloride (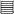) or with chloroquine (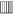). Results observed after either nystatin or amiloride incubations were not significantly different for all the densities. After chloroquine treatment, significant decreases of E-[c(RGDfK)2] uptake were presented at low, medium and high density (39.8%±2.6%; 38.8±0.8%; 29.5%±2.4%, respectively). Significant iRGD uptake decreases were observed when cells were incubated with chloroquine, 42.4%±1.1% at low density, 48.2±0.2% at medium density and 53.9%±2.5% at high density. The level of autofluorescence of the cells was represented by the dotted line (……). For these experiments (n≥3), the results (☆) and (★,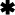,•,
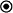
○) were significantly different with p<0.05 and p<0.01, respectively. Insert part B, fluorescent microscopy of cells treated with chloroquine, showed a mainly cytoplasmic membrane signal of iRGD (arrows).

The E-[c(RGDfK)2] and iRGD ligand accumulation inside the cells is known to follow integrin-dependent and/or independent fluid phase endocytosis mechanisms. The characterization of the caveolin, macropinocytosis and clathrin roles and their potential cell density dependence were studied using nystatin, chloroquine or amiloride (figure 4). These compounds inhibit the caveolin-dependent endocytosis, the clathrin-dependent endocytosis and the macropinocytosis, respectively.

Inhibition of the caveolin-dependent endocytosis did not alter the internalization of the E-[c(RGDfK)2] or iRGD ligands for the studied cell densities. Similarly, macropinocytosis inhibition had no effect on the E-[c(RGDfK)2] and iRGD internalizations.

The internalization of both E-[c(RGDfK)2] and iRGD were significantly altered during inhibition of the clathrin-dependent endocytosis by chloroquine regardless of the cell density. Nevertheless the clathrin involvement did not appear similar for E-[c(RGDfK)2] and iRGD internalizations. When the cell density increased, the inhibition of the E-[c(RGDfK)2] ligand internalization by the chloroquine decreased from 40% to 29%. The iRGD internalization was also sensitive to chloroquine but, as opposed to E-[c(RGDfK)2], the inhibition increased significantly from 42% to 56% when the cell population density increased.

Fluorescence microscopy showed a typical cytoplasmic membrane labeling when iRGD ligand incubations were performed during the inhibition of the clathrin-mediated endocytosis (insert figure 4B). Moreover, in this condition, RGD ligands bound to the plasma membrane represented the major part of the cytometric signal. Interestingly, when cells were in the presence or not of nystatin, the fluorescence location of the E-[c(RGDfK)2] or iRGD ligands was similar, inside cytoplasmic vesicles (data not shown).

### Role of microtubules and actin filaments on the internalization of E-[c(RGDfK)2]-Alexa488 and iRGD-alexa488 peptides at different cell densities

**Figure 5:**
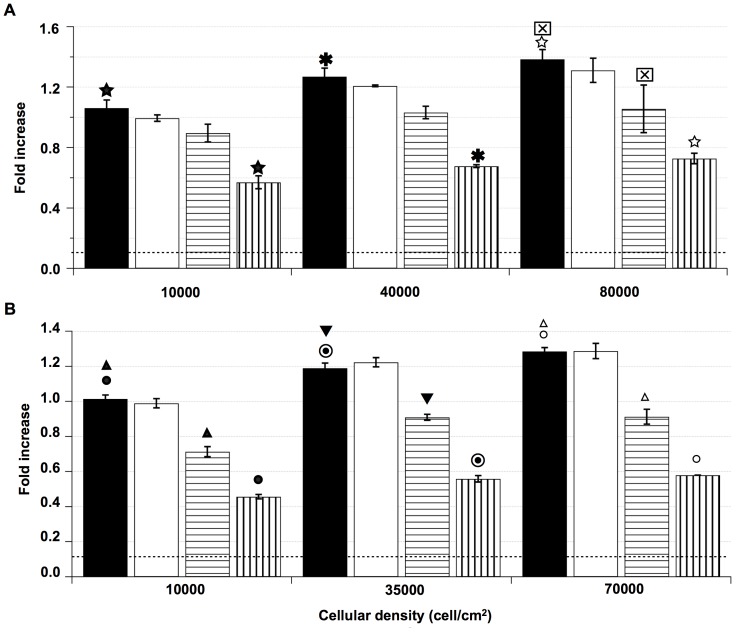
Cellular density effect on the cytoskeleton contribution to E-[c(RGDfK)2] and iRGD internalization. U87 cells were incubated with E-[c(RGDfK)2] at different densities (10000; 40000; 80000 cell/cm^2^) (A) or with iRGD (10000; 35000; 70000 cell/cm^2^) (B). These densities were defined as low, medium and high density, respectively. Incubations were performed without inhibitor (▪), with paclitaxel (□), with cytoD (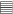) or with colchicine (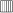). Results with paclitaxel were not significantly different. The cytoD effect on the E-[c(RGDfK)2] internalization was not significant at low density (15.65%±1.3%) but appeared significant for the highest density (23.9%±7.9%). For iRGD, significant results were observed for all the densities when cells were treated with cytoD (29.7%±1.3%; 23.5%±1.3%; 29.0%±2.1% at low, medium and high density, respectively). Treatment with colchicine induced a strong decrease of E-[c(RGDfK)2] and iRGD uptakes. For E-[c(RGDfK)2] the uptake results were 46.3%±1.3%, 46.6±3.0, and 47.5%±3.0% at low, medium and high density, respectively. iRGD uptake results were of 55.0%±0.3%, 53.1%±0.1% and 54.8%±0.8% at low, medium and high density, respectively. For these experiments (n≥3), the results (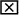) and (★,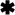,☆,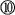,▾,▵,•,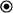,○) were significantly different with p<0.05 and p<0.01, respectively.

Endocytosis pathways and intracellular movements of the integrins are highly regulated by the balance between the polymerized and unpolymerized states of actin and microtubules. Thus, its effect on the internalization of E-[c(RGDfK)2] and iRGD was assessed for various cell densities (figure 5).

The alkaloid cytoD is a reversible inhibitor of the actin polymerization that binds to F-actin [15] and generates an immediate disruption of actin filaments. Incubations of U87 cells with cytoD revealed only a low contribution of actin in the processes of E-[c(RGDfK)2] internalization being only significant for the highest cell density. iRGD uptake was significantly more sensitive to actin depolymerization by cytoD and the observed inhibition was not altered by the cell density.

The possible role of microtubules was studied using either paclitaxel or colchicine. Paclitaxel (taxol) binds β-tubulin and blocks the microtubules in a polymerized state [16]. Colchicine is an alkaloid that induces a reversible depolymerization of the tubulin [17].

Stabilization of polymerized microtubules by paclitaxel did not alter E-[c(RGDfK)2] nor iRGD ligand internalizations. However, after the colchicine induced depolymerization of microtubules, both the E-[c(RGDfK)2] and the iRGD internalizations were reduced by 47% and 50%, respectively, at all cell densities. U87 cells treatment with nocodazole, another microtubule depolymerization agent [18], showed similar results (data not shown).

### Cell energy status, for various U87 cell densities, modulated the E-[c(RGDfK)2]-Alexa488 and iRGD-alexa488 uptakes

Clathrin-dependent endocytosis, cytoskeleton regulation and intracellular traffic are active mechanisms and require the contribution of ATP. The main energy pathways involved in the RGD ligand internalization were investigated at several cellular densities.

**Figure 6:**
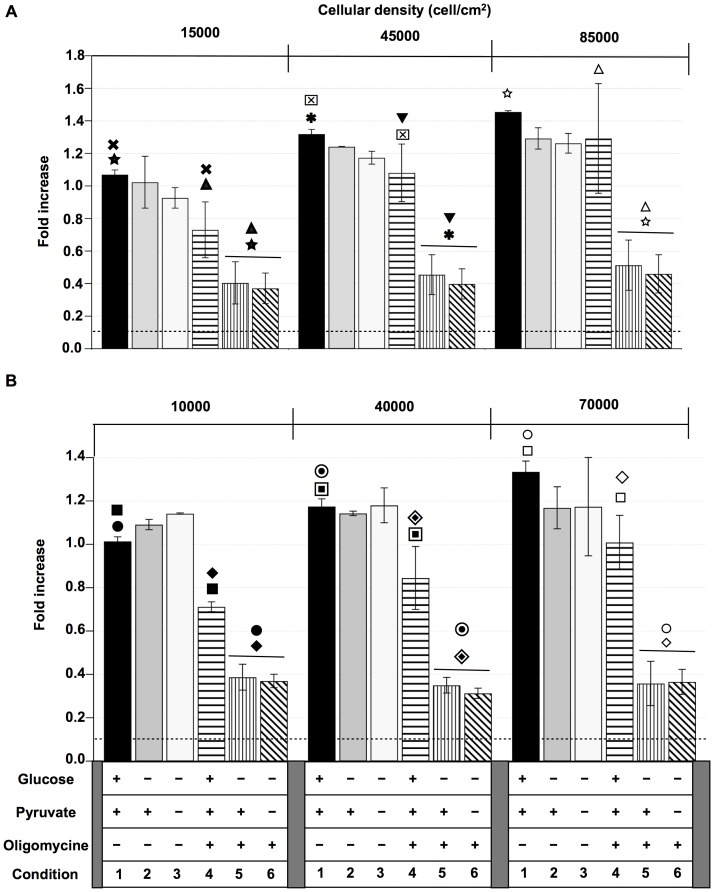
Metabolism contribution to E-[c(RGDfK)2] or iRGD internalizations: Effect of the cellular density. U87 cells were incubated for 1-[c(RGDfK)2] (A) and iRGD (B). Incubations were performed for different media conditions: with Glucose and pyruvate, without oligomycin (condition 1); with Pyruvate, without Glucose and oligomycin (condition 2); without Glucose, pyruvate and oligomycin (condition 3); with Glucose, pyruvate and oligomycin (condition 4); with pyruvate and oligomycin, without Glucose (condition 5); with oligomycin, without Glucose and pyruvate (condition 6). All the experiments were performed at least 3 times. For the E-[c(RGDfK)2] the results for all the conditions (★,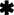,☆,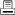,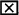,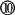,▾,▵) were significantly different with p<0.01. For the iRGD the results for all the conditions (▪,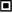,□,•,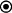,○,♦,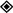,⋄) were significantly different with p<0.01.

The U87 cells were pre-incubated with different media containing or not pyruvate or D-glucose and in the presence or not of inhibitors of the oxidative metabolism (figure 6).

When cells were incubated without glucose and with or without pyruvate (conditions 2 and 3) the E-[c(RGDfK)2] or iRGD ligand internalizations were not altered and remained cell density dependent. When the oxidative metabolism was inhibited by oligomycin, an ATP synthase inhibitor, in presence of glucose and pyruvate (condition 4), the internalizations of E-[c(RGDfK)2] or iRGD ligands decreased more for the lowest cell densities than for the highest ones. Incubation without glucose in the presence of pyruvate and oligomycin (condition 5) showed a strong inhibition of E-[c(RGDfK)2] or iRGD internalizations regardless of the cell density. Similar results were observed when cells were also pyruvate deprived (condition 6). When experiments were performed with rotenone to inhibit the complex I of the respiratory chain, the results were comparable to the ones realized with oligomycin (data not shown).

### MRI of E-[c(RGDfK)2]-DOTA-Gd internalized by cells at various densities

**Figure 7:**
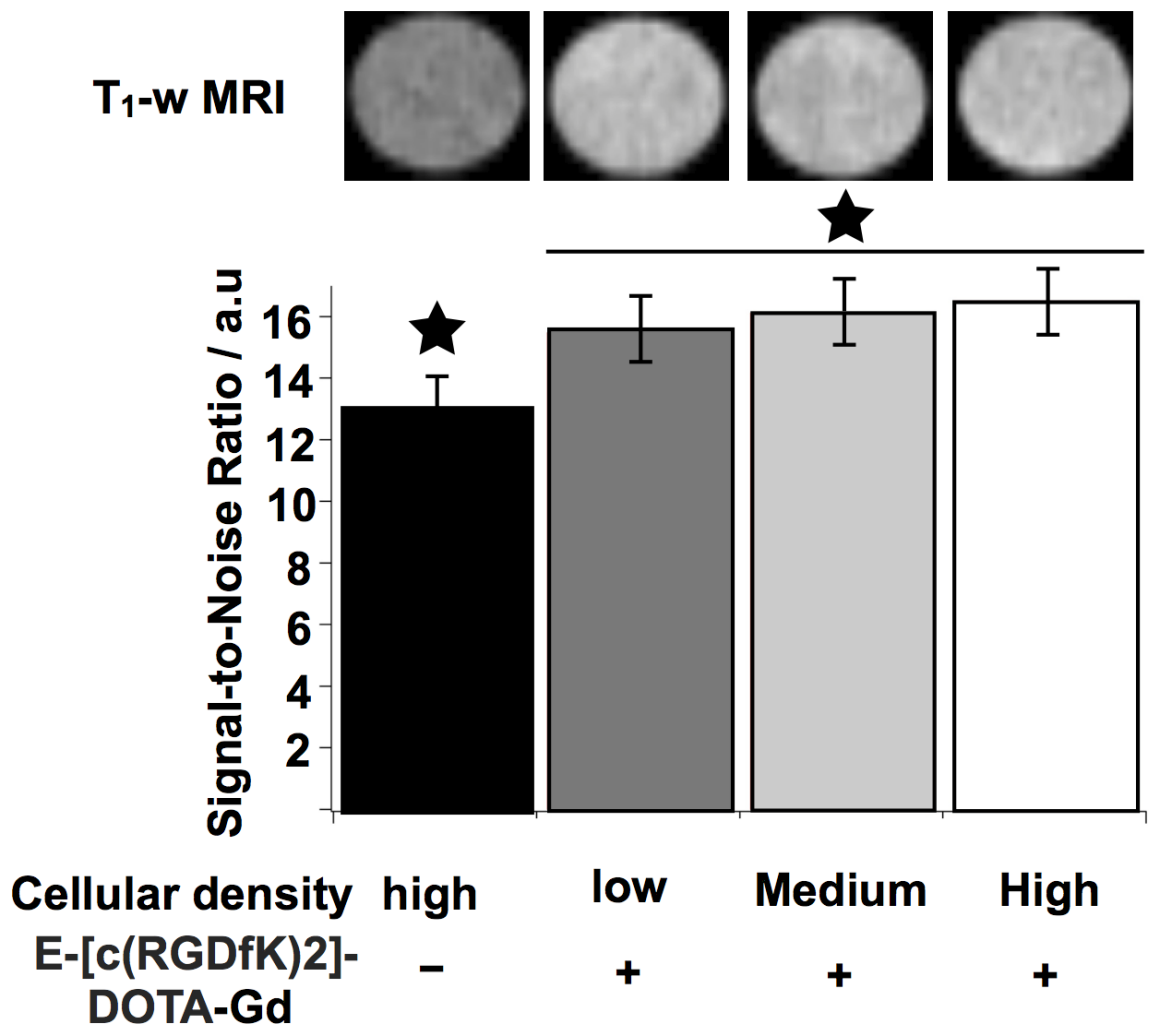
MRI charaterization of the cellular density effect on the E-[c(RGDfK)2]-DOTA-Gd internalization. U87 cells were incubated, for different densities, with E-[c(RGDfK)2]-DOTA-Gd (400 µM). SNR of the different cell densities were significantly higher than the SNR of control (★, p<0.05). The SNR values for the low, medium and high density were not significantly different.

Fluorescence experiments showed that the internalization of RGD peptides was dependent on the cellular density. MRI signal could also be correlated to tumor cell density and could consequently improve diagnosis. U87 cells, seeded at various cellular densities, were incubated with E-[c(RGDfK)2]-DOTA-Gd. MR images of these cells showed a SNR enhancement compared to the unlabeled control cells. The SNR increase appeared cell density dependent being 15.5 and 16.5 for the lowest and the highest densities respectively (figure 7). Although, this variation was in the range of the experimental errors, a trend was visible opening the door to the exploitation of this variation with more sensitive imaging method associated to the RGD ligands.

## Discussion

Cell density is a first messenger that regulates the cell signaling from receptor trafficking [19] until transcriptional control steps [20]. Numerous multiple modalities like protein translocation between cellular compartments including the cytoplasmic membrane, cytosolic organelles and the nucleus are involved. In the desensitization processes, the receptor internalization is also under the control of the cell growth by contact inhibition. For instance, EGFR and VEGFR-2 internalizations and the frizzled-7 turnover are affected by the cell density [21]–[23].

In our study, we demonstrated that the RGD and iRGD motif internalizations appeared dependent on cellular density for U87 cells that have lost cell contact inhibition and are characterized by a strong invasiveness. Interestingly, in this glioblastoma model, the αvβ3 level expression is not regulated by the cell density (data not shown). This is a new form of a cell signaling regulation of internalization process involving the integrin αvβ3. Previous correlations between tumor grade, infiltrative and invasive areas with the αvβ3 over-expression have been demonstrated [24], [25]. In this U87 cell model, the RGD ligand internalization study revealed the regulation of steps downstream from αvβ3 engagement.

Therefore the characterization of RGD internalization processes could be helpful to develop new concepts and/or new agents allowing the assessment of tumor cell division potential with more accuracy in terms of both location and cell invasiveness quantification.

Hence, the internalization properties of the RGD ligands were initially studied by the linkage of RGD with MRI contrast agents with the purpose to correlate quantitatively the MRI SNR with the cell density within specific areas of the glioblastoma. Fast RGD internalization was previously described for a concentration similar to our study [11]. Moreover, incubating Jurkat cells with a mimetic RGD linked to Gd at the same concentration, Burtea et al. demonstrate a significant MRI signal enhancement [26]. In vivo, these auteurs showed a half-life clearance near about 1 h, sufficient to image atherosclerotic plaques. This time of life is expected to be long enough to obtain detectable internalization of RGD peptides by the tumor cell vasculature and to mesure the cellular density in glioma. Nevertheless, in our study, MR images displayed a low variation of the RGD-Gd labeled cells SNR when the cell density increased in vitro. Consequently, the lack of MRI sensitivity remains a limiting factor to perform quantitative imaging. This fact highlights the necessity to develop other concepts like dynamic nuclear polarization enhanced MRI [27], [28] with adapted contrast agents. The latter could be able to target the elements of the integrin internalization process and to improve the MRI sensitivity without altering MRI spatial resolution. Moreover, combination of MRI with imaging techniques like positron emission tomography (PET) could offer simultaneously the resolution and the sensitivity to characterize the cell density that correlate with tumor cell invasion. Indeed, the magnitude of ^11^C-methionine accumulation has been shown to reflect tumor cell density but the images do not correlate to the tumor infiltrated areas [29]. The ^18^F-FDG-^11^C-methionine uptakes, described by Kinoshita et al., appear as better indicators of the tumor infiltrated brain edema although the decoupling score remains dependent on anatomic fluctuation [30]. Thus, quantification of the cell density remains an open challenge in the development of new tools allowing straightforward quantitative relationships between the cell density and the corresponding signal.

Likewise, a better understanding of the transduction steps concerned in the RGD internalization may point out new cell density targets for imaging.

Clathrin-dependent endocytosis is described as being involved in the internalization of integrins and their ligands. In our model this endocytosis pathway played an important role in the internalization of E-[c(RGDfK)2] and iRGD peptides. For E-[c(RGDfK)2], the contribution of clathrin decreased with the rise of cell density. An opposite result was obtained for iRGD. This difference of behavior can be explained by the possible interactions between the intregrin internalization processes and the neuropillin-1 receptors as previously described [31]. Actin interacts with clathrin and the integrins [32] [33] [34]. In our model, actin was involved in the iRGD internalization and only partially for E-[c(RGDfK)2] one. Microtubules are described to be involved in the clathrin dependent endocytosis [35] [36]. Here, we gave evidences that microtubules played a major role compared to actin in the E-[c(RGDfK)2] and iRGD internalizations but without cellular density contribution.

Endocytosis and transport of intracellular vesicles are under the control of multiple signaling pathways. Different small GTPases such as ROCK-A, Dynamin, and others have a key role in these processes [36] [37] [38] [39]. Inhibition of ROCK and dynamin-2 by Y-27632 dihydrochloride and MITMAB inhibitor respectively did not alter E-[c(RGDfK)2] internalization (data not shown). Dynamin-1 can compensate the dynamin-2 inhibition as dynamin-1 is known being typically expressed in neurons, neuroendocrins cells and also in glioma cells [40]. The regulation of integrin activity requires phosphorylation by kinases, like the focal adhesion kinases (FAK) [41]. In our model, inhibition of FAK by PF-573228 had little effect on the RGD internalization (data not shown).

Phosphorylations are also described as key steps in the regulation of internalization and transport processes. These phenomena need an ATP supply by the cell metabolism. The contribution of the major metabolic pathways on the internalization of E-[c(RGDfK)2] or iRGD were investigated. In all conditions, the glycolytic pathway appeared as the major process that supplied the energy for internalization of RGD-ligands. This contribution increased with the cell density. When cells were deprived of glucose as the main glycolysis substrate, they were able to compensate using the oxidative phosphorylations. Moreover, this shift did not alter the contribution of the cell density to modulate the RGD internalization. Oxidative metabolism played a minor role with its highest level at the lowest cell densities. Analysis of the experiments performed without glucose, pyruvate and oligomycin suggested that the products from the oxidative degradation of amino-acids and from the beta oxidation of fatty acids could be a source of substrates for the TCA cycle and the electron chain transport. In these conditions, the energetic status was high enough to allow the RGD-ligand internalization. A low uptake of RGD-peptides was observed when cells were incubated without glucose in the presence of oligomycin with or without pyruvate. This observation suggests the contribution of other metabolic pathways. The glycogenolysis and/or the anaplerotic pathways using glutamine are able to supply ATP in order to obtain a minimal internalization of RGD peptides. Nevertheless the level of fluorescence could be also representative of RGD peptides bound to the cell membranes. Tumor metabolism has been described to be dependent on tumor type, growth dependent modifications of tumor cell phenotypes and the age of the tumor cells [42] [43]. Thus, the metabolism shows an important plasticity and modulates the energy dependent processes. In our study, the modulation of the RGD-peptide internalization by the cell density with a high glycolytic energetic pathway involvement was in agreement with the invasiveness and/or the grade of the tumor [44]. Moreover the balance between the glycolytic metabolism and the oxidative phosphorylations evidenced the glioma metabolic heterogeneity that also correlates with the invasiveness and/or the grade.

This study characterized the internalization pathways of E-[c(RGDfK)2] or iRGD looking at their accumulation in cells and some properties of the intracellular vesicle transports as a function of the cell density. Others parameters involved in RGD internalization, and potentially regulated by the cell density, like the contributions of others integrins and modulations of the αvβ3 affinity for RGD must not be underestimated. However, we demonstrated that the integrin expression level is not the only element that can increased the RGD uptake in proliferative glioblastoma cells. Indeed the transduction pathways of the integrin internalization can modulate this uptake. Moreover, the various compartments of these pathways are affected by the cell density. This latter property could be used for the development of new probes targeting these elements with the purpose to design a tool for a quantitative evaluation of the glioma cellular density by MRI.
